# Intein-mediated backbone cyclization of VP1 protein enhanced protection of CVB3-induced viral myocarditis

**DOI:** 10.1038/srep41485

**Published:** 2017-02-02

**Authors:** Xingmei Qi, Sidong Xiong

**Affiliations:** 1 Jiangsu Key Laboratory of Infection and Immunity, Institutes of Biology and Medical Sciences, Soochow University, Suzhou, Jiangsu 215123, China

## Abstract

CVB3 is a common human pathogen to be highly lethal to newborns and causes viral myocarditis and pancreatitis in adults. However, there is no vaccine available for clinical use. CVB3 capsid protein VP1 is an immunodominant structural protein, containing several B- and T-cell epitopes. However, immunization of mice with VP1 protein is ineffective. Cyclization of peptide is commonly used to improve their *in vivo* stability and biological activity. Here, we designed and synthesizd cyclic VP1 protein by using engineered split *Rma* DnaB intein and the cyclization efficiency was 100% in *E. coli*. As a result, the cyclic VP1 was significantly more stable against irreversible aggregation upon heating and against carboxypeptidase *in vitro* and the degradation rate was more slowly *in vivo*. Compared with linear VP1, immunization mice with circular VP1 significantly increased CVB3-specific serum IgG level and augmented CVB3-specific cellular immune responses, consequently afforded better protection against CVB3-induced viral myocarditis. The cyclic VP1 may be a novel candidate protein vaccine for preventing CVB3 infection and similar approaches could be employed to a variety of protein vaccines to enhance their protection effect.

Coxsackievirus B3 (CVB3) is an important human pathogen that induces acute and chronic viral myocarditis in children and young adults^1^. About 5–50% of myocarditis and its end stage, dilated cardiomyopathy, is attributable to CVB3 infection^2^,^3^. However, there is currently no vaccine or therapeutic reagent in clinical use, although some reports have demonstrated effective vaccination strategies for CVB3 in animal models. These include inactivated virus, attenuated live virus, DNA vaccines, and virus-like particle (VLP) vaccines^4^,^5^,^6^,^7^,^8^,^9^,^10^,^11^,^12^,^13^,^14^,^15^,^16^. These approaches have not been applied clinically because of safety concerns. For example, attenuated and inactivated virus vaccines carry the risks of reversion to a virulent form or incomplete inactivation^17^. As a theoretically technology choice for DNA vaccine are underway on the clinical trials for a variety of infectious diseases and cancers, while the scientific basis of DNA vaccines has yet to be clearly defined. Proteins can be good alternatives to vaccines, because the degradation products are simply amino acids and would not have toxicity. Moreover, recombinant protein vaccines are much safer containing purified antigens with defined structures and can induce both cellular and humoral immune response^4^,^18^.

CVB3 capsid protein VP1 is an immunodominant structural protein, containing several B- and T-cell epitopes^19^. It has been reported that vaccinated with a recombinant plasmid encoding VP1 gene of CVB3 was shown to confer protection against lethal CVB3 infection in mice^6^,^8^,^9^. However, immunization of mice with a recombinant VP1 protein is ineffective due to antigen presentation to the immune system being ineffective^4^. Also, the termini of linear proteins are often flexible and easily get degraded rapidly by proteolytic enzymes^20^,^21^. Studies have shown that the cyclization of flexible linear peptides frequently improve their *in vivo* stability and biological activity due to the conformational rigidity and the resistance to hydrolysis by exopeptidases by lacking of both amino and carboxyl termini^20^,^22^,^23^. These highly stable backbone cyclization small peptides have been widely used in a range of new applications, such as medicinal chemistry to improve the biochemical and biophysical properties of flexible and labile peptides in the development of peptide-based drug candidates^24^,^25^,^26^ and provide biologists with a powerful molecular tool^27^,^28^. The *trans*-splicing ability of split intein has been used in protein engineering to efficient *in vivo* and *in vitro* cyclization of large proteins and small peptides. The use of protein *trans*-splicing (PTS) mediated backbone cyclization for the biosynthesis of circular polypeptides is of special interest, since it would proceed during expression of the fusion gene in living cells, thereby offering a time saving and economical approach. Several large proteins have been successful cyclized through protein *trans*-splicing. For example, the artificially split intein *DnaB* from the *Synechocystis sp. PCC6803 (Ssp DnaB*) strain was used for the cyclization of the TEM-1 β-lactamase in the bacterial periplasm^29^, the naturally split intein from the dnaE gene of Synechocystis sp. PCC6803 (*Ssp DnaE*) was employed to cyclize a mutant of GFP protein^30^ and dihydrofolate reductase^31^. These circular proteins were more thermostable than the linear form and significantly more resistant to proteolysis of exopeptidase. However, backbone cyclized proteins as a potential protein vaccine to enhance immune responses have not been explored. Moreover, the inteins used to polypeptides backbone cyclization were not efficitive, which typically leads to heterogeneous products with low yield^29^,^30^,^31^,^32^,^33^.

In this study, we used an artificial split intein from the *dnaB* gene of *Rhodothermus marinus (Rma DnaB*) to generate a split functional N- and C- terminal intein to cyclize the VP1 protein of CVB3 in *E. coli*. The cyclization reaction was achieved by sandwiching VP1 contained a H_6_-tag between the C-terminal 43-residue segment (RB_C_) and N-terminal 104-residue segment (RB_N_) of the *Rma DnaB* intein in the permutated order RB_C_-VP1-RB_N_. This design successfully produced the VP1 protein with a circular topology, in which the N- and C-termini are cross-linked with a normal peptide bond (Fig. 1a) and the cyclization efficiency is about 100%. We characterized the stability and biological activity of the cyclic VP1 protein and tested further for its *in vivo* efficiency of inducing CVB3-specific immune responses and protection mice against CVB3-induced myocarditis as a potential anti-CVB3 protein vaccine.

## Results

### Preparation of circular VP1

**Figure 1:**
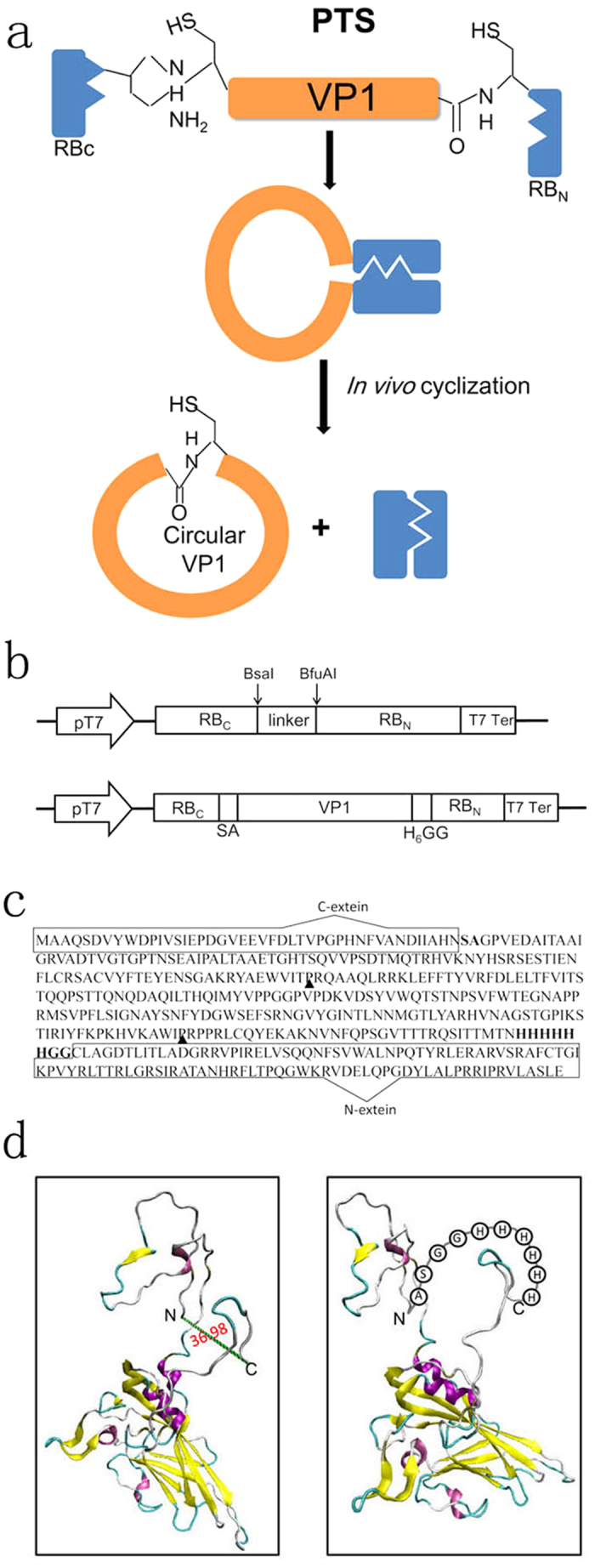
Protein cyclization *in vivo* using intramolecular *trans*-splicing activity of *Rma DnaB* intein. (**a**) VP1 is sandwiched between the C-terminal fragment (RB_C_) and N-terminal fragment (RB_N_) of *Rma DnaB* intein. Splicing mediates the ligation of the N and C termini of VP1 through a native peptide bond. (**b**) Schematic representation of expression vector pERB_C_-RB_N_ and pERB_C_-VP1-RB_N_. (**c**) Amino acid sequence of the fusion protein RBc-VP1-RB_N._ The C-terminal 43-residue segment (RB_C_) and the N-terminal 104-residue segment (RB_N_) of the *Rma DnaB* intein are enclosed with a rectangle. The linker sequence of SA and H_6_-GG is bold and the thrombin-specific cleavage sites marked with black arrow. (**d**) Models of the 3-D structure of linear (left) and circular form of VP1. The models were created based on the coordinates of the PDB code 1COV. N and C indicate N- and C-termini in the linear form.

The split intein *trans*-splicing requires specific amino acid residues at the intein-extein junctions for efficient protein splicing. Thereby, the entire vp1 gene was sandwiched between the C- and N-terminal fragments (I_C_ and I_N_) of *Rma DnaB* intein with SA residues at its N terminus and GG residues at its C terminus (Fig. 1a–d). Meanwhile, the N- and C-termini of VP1 protein in 3-D structure are in close proximity (36.98 Å), which also facilitates the protein backbone cyclization (Fig. 1d, left). To facilitate protein purification, a histidine tag was added at the 3′ end of VP1 protein. After cyclization, the original N- and C-termini of VP1 would then be connected by a loop of an additional 10 amino acids (Fig. 1d, right).

**Figure 2:**
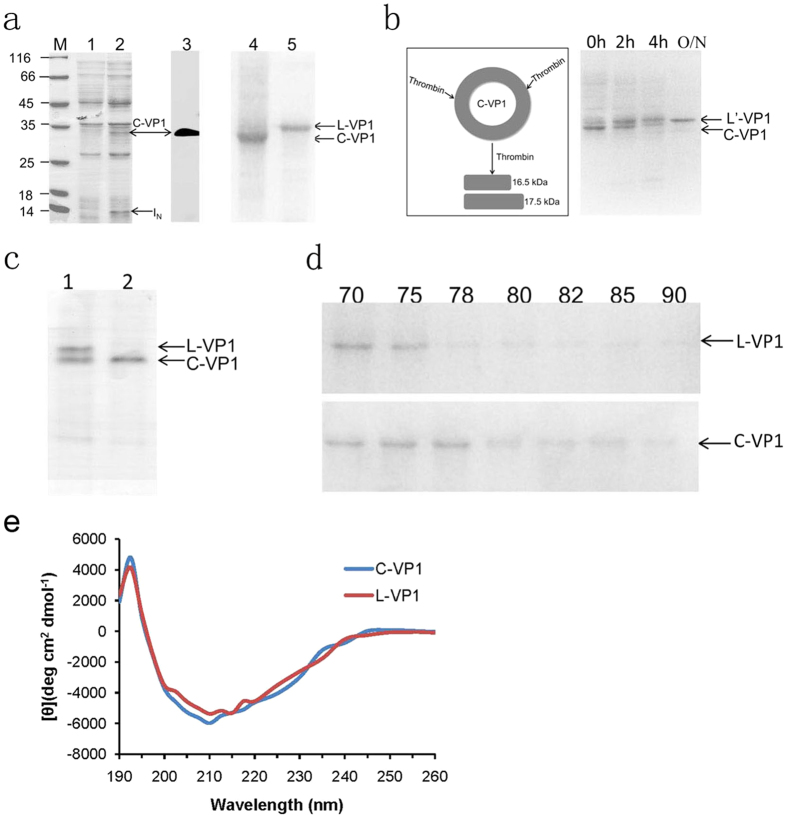
Protein expression, purification and characterization. (**a**) SDS-PAGE and Western blotting analysis of VP1 protein expression. Protein bands were visualized by Coomassie Blue staining (lane 1, 2, 4, 5) or by Western blotting (lane 3). Lane M, protein marker; Lane 1, total cellular proteins of *E. coli* before IPTG-induced expression; lane 2, total cellular proteins of *E. coli* after IPTG-induced expression at 37 for 4 h; lane 3, protein bands were visualized by Western blotting using anti-VP1 antibody; lane 4, purified cyclic VP1 (C-VP1); lane 5, purified linear VP1 (L-VP1). (**b**) Diagram illustrating the proteolytic treatment on C-VP1 by thrombin and time course of the digestion. 0 h: before addition of thrombin; 2 h, 4 h, O/N: time after addition of thrombin. The positions of the circular and linear form of VP1 are indicated. (**c**) Proteolysis resistance of C-VP1. Lane 1, L-VP1 and C-VP1 mix proteins before addition of carboxypeptidase Y; Lane 2, incubation of L-VP1 and C-VP1 mix proteins with carboxypeptidase Y at 25 °C for 10 h. (**d**) Effect of heat treatment at different temperatures on C-VP1 and L-VP1. C-VP1 stands for splicing product of cyclic VP1 protein, I_N_ stands for N-terminal fragment of *Rma DnaB*, L-VP1 stands for linear VP1 protein, L′-VP1 stands for cyclic VP1 digestion by thrombin. (**e**) CD spectra of L-VP1 and C-VP1 protein.

VP1 cyclization was readily apparent by SDS-PAGE on IPTG induction of pERB_C_-VP1-RB_N_ (Fig. 2a, lane 2). Bands with apparent molecular masses corresponding to the cyclic VP1 (C-VP1, 32 kDa) and I_N_ (13 kDa) products were clearly visible, while the I_C_ (3 kDa) fragment was too small to detected on SDS-PAGE. The fusion protein RB_C_-VP1-RB_N_ (50 kDa) and cleavage products (RB_C_-VP1, 37 kDa or VP1-RB_N,_ 46 kDa) were not visible on SDS-PAGE. IPTG-induced sample from lane 2 was further confirmed by Western blotting with an anti-VP1 antibody (Fig. 2a, lane 3), which indicated the presence of only cyclic VP1 protein band. The result indicated that the *trans*-splicing activity of *Rma DnaB* intein for VP1 protein cyclization was completely and the *in vivo* protein cleavage was not observed. Purified C-VP1 and L-VP1 proteins were obtained as described in material and method and verified by SDS-PAGE. The C-VP1 migrated more rapidly in SDS-PAGE analyses than did L-VP1 (34 kDa, Fig. 2a, lane 4 and 5), implying an additional topological constraint.

### Characterization of C-VP1 protein

The successful cyclization of the VP1 protein was confirmed by re-linearization by limited proteolysis. It speculated two thrombin-specific cleavage sites in the amino acid sequence of VP1 protein by Peptide Cutter as indicated in Fig. 1d (http://web.expasy.org/peptide_cutter/). Then treatment by thrombin, the C-VP1 should be cut into two polypeptides (Fig. 2b). However, the digestion of cyclic VP1 by thrombin resulted in a liner form of VP1 (L′-VP1) not two polypeptides, which also migrates more slowly than the circular form. It is possible that the two theoretical thrombin-specific cleavage sites were only cut one by thrombin. The disappearance of the lower band (circular form) and the re-appearance of the upper band (linear form) after thrombin digestion thus indicates that cyclic VP1 could be re-linearized into the linear form by limited proteolysis. Since circular proteins lack both N- and C-termini, they should be increasing the stability and resistance to exopeptidase. Hence, we tested the sensitivity against carboxypeptidase Y. SDS-PAGE showed that L-VP1 was degraded by proteolytic cleavage, while the C-VP1 exhibited no degradation under the same conditions, suggesting that the termini of this VP1 protein was joined via a peptide bond, conferring resistance to exopeptidase treatment (Fig. 2c).

The cross-linking of polymer chains will reduce the conformational entropy of their unfolded state, resulting in stabilization of the native state [3]. To test resistance against heat precipitation, the linear and circular form of VP1 proteins were incubated at various temperatures for 30 min (from 70 °C to 90 °C). Then, remove the precipitate by centrifugation and analyze the soluble fraction by SDS-PAGE. As shown in Fig. 2d, at 78 °C, nearly 100% of the linear form (indicated as L-VP1) precipitated, while the circular form (indicated as C-VP1) of VP1 entirely denatured at 80 °C, indicating a stability enhancement of at least 2 °C by cyclization. The data indicate that cyclization enhanced the thermostability of VP1.

We also investigated the effect of the backbone cyclization on the secondary structure of VP1 protein by using CD spectroscopy. As shown in Fig. 2e, the backbone fold structure is similar in both linear and circular form VP1, which indicated that the backbone cyclization didn’t change the overall three-dimensional structure of the VP1 protein.

### Vaccination of mice with C-VP1 elicits strong humoral and cellular immunity

**Figure 3:**
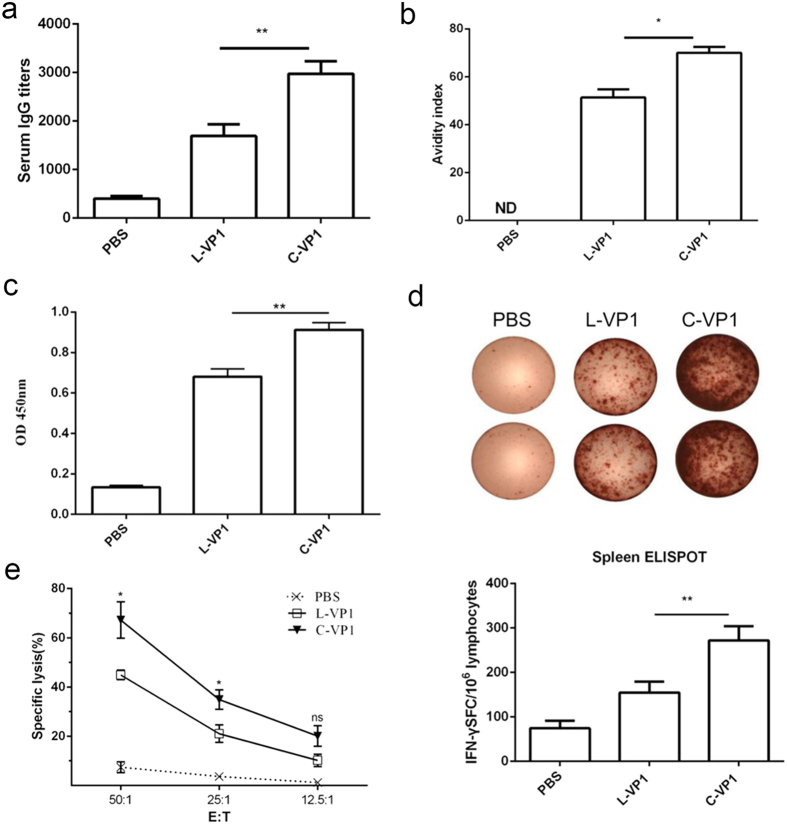
CVB3-specific systermic responses were elicited by immunization with groups of C-VP1, L-VP1 or PBS administration. (**a**) CVB3-specific IgG levels were measured by ELISA 2 weeks after the last immunization, (**b**) antibody avidity. (**c**) CVB3-specific T cell proliferation was assessed by Roche BrdU-Kit after stimulation with 20 ug/ml VP1_237–249_ peptide in the culture of 20 U/ml IL-2 for 72 h. (**d**) CVB3-specific cytokine-secreting lymphocytes were quantified by ELISPOT assay in response to VP1_237–249_ peptide. (**e**) CVB3-specific CTL activity of splenic cells was evaluated by lactate dehydrogenase assays using pcDNA3.1-VP1 stable transfected autologous SP2/0 cells as target cells. Individual experiments were repeated 3 times with similar results, *P < 0.05, **P < 0.01. ND, not detected.

BALB/c mice were vaccinated with C-VP1 3 times biweekly, and CVB3-specific IgG and T cell immune response were evaluated 2 weeks following the last immunization. To assess humoral immune responses of the different protein vaccine, serum samples were collected and titers of total anti-CVB3-specific IgG were determined by ELISA. As shown in Fig. 3a, mice immunized with C-VP1 exhibited the maximal titer of 1:2974 compared to 1:1691 of L-VP1 immunized mice. Meanwhile, the avidity of the antibody was also evaluated as shown in Fig. 3b. The avidity indices of serum IgG elicited by C-VP1 was 70, significantly higher than those of L-VP1 immunized mice (51.3). These data clearly indicated that immunization with C-VP1 can efficiently induce anti-CVB3 antibody production.

To evaluate the ability of C-VP1 to induce CVB3-specific spleen T cell immune response, T cell proliferation and IFN-γ-producing T cell frequency in spleen were evaluated. As shown in Fig. 3c, compared with mice given PBS or L-VP1, mice immunized with C-VP1 elicited a significant increase in specific T cell proliferation response. More importantly, significantly increased frequency of IFN-γ-producing T cells was also observed in the spleen of C-VP1 group (272 vs. 154 SFC/10^6^ cells, Fig. 3d). In agreement with the ELISPOT data, CVB3-specific CTLs activity of spleen cells obtained from C-VP1 immunized mice were 67.24% with at an E:T ratio of 50:1, which was significantly higher than that from L-VP1 immunized mice (43.37%, Fig. 3e). Taken together, these data indicate that C-VP1 vaccines can also induce a strong antigen-specific T cell immune response.

### Increased half-life of C-VP1 facilitated dendritic cell (DC) maturation

**Figure 4:**
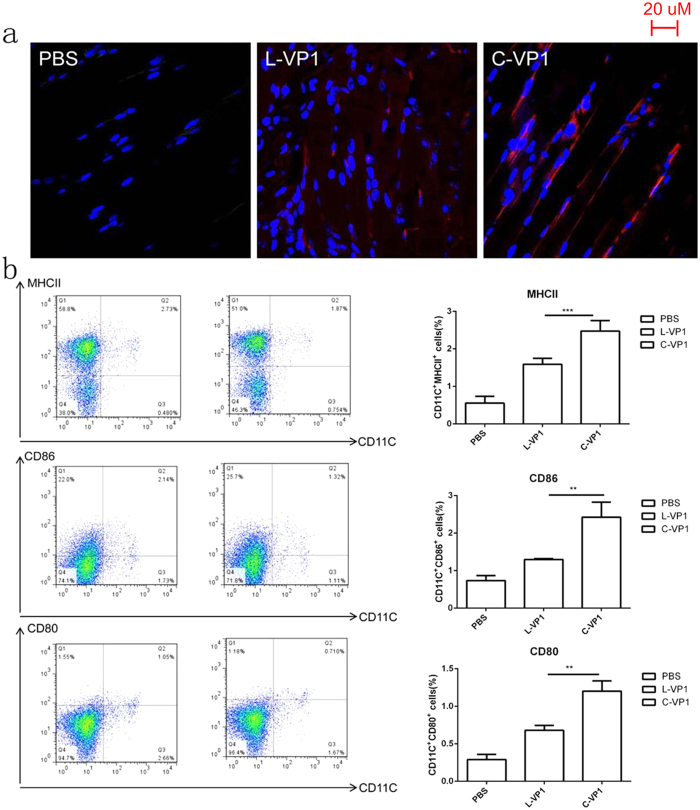
Validation of C-VP1 stability *in vivo* and spleen DCs maturation. Mice were intramuscularly immunized with 25 ug of purified C-VP1or L-VP1 protein, four days later (**a**) muscles were collected and subjected to Immunohistochemical analysis to detect protein stability *in vivo* and (**b**) spleens were collected and stained with DC maturation markers CD80, CD86 and MHCII to determine by flow cytometry, **P < 0.01, ***P < 0.001.

To further explore the underlying mechanism of C-VP1 in improving immune responses against CVB3-induced myocarditis, we analyzed its stability *in vivo* by Immunofluorescence staining of VP1 protein. As shown in Fig. 4a, compared with L-VP1, the degradation rate of C-VP1 protein was much more slowly at the sites of immunization. In accordance with the stabilization effect of the polypeptide backbone cyclization, the increased maturation of spleen DC in C-VP1 immunized mice was observed. The results showed that immunization of mice with C-VP1expressed higher levels of MHCII, CD80 and CD86 than L-VP1 immunization mice (Fig. 4b). There was an approximately 1.5-fold increase in cell surface MHCII expression, a 1.8-fold increase in CD80 expression, and a 1.9-fold increase in CD86 expression in mDC upon immunization with C-VP1. These result indicate that mice immunized with C-VP1 induced mDC maturation more effectively than L-VP1 immunization mice.

### Enhanced protection against viral myocarditis by C-VP1 vaccination

**Figure 5:**
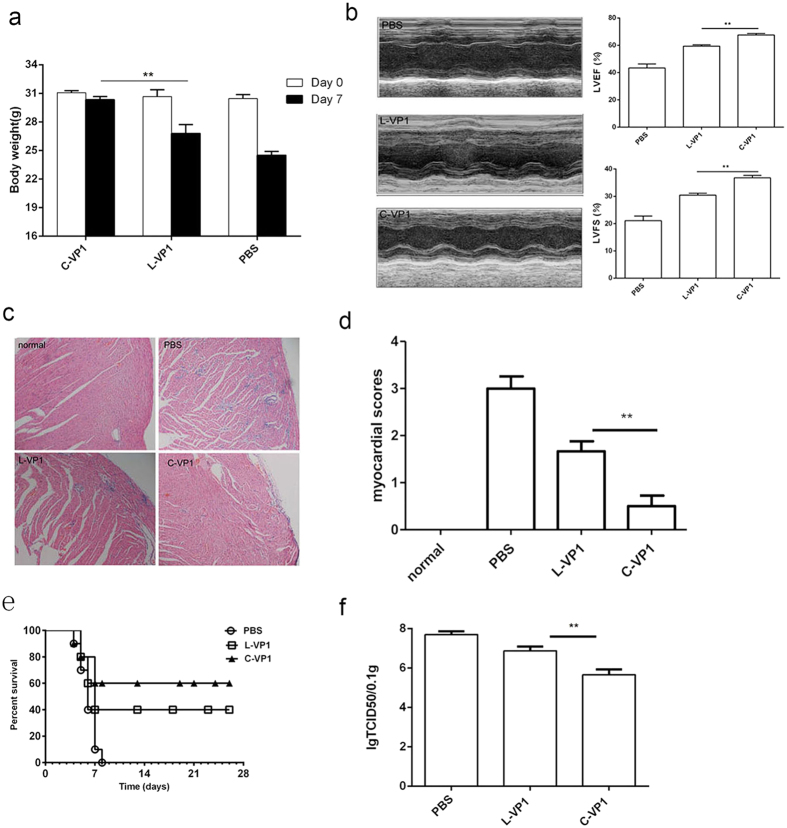
Enhanced resistance to CVB3-induced acute myocarditis by immunization with C-VP1. Seven days after 3LD50 CVB3 challenge. (**a**) Body weight loss. (**b**) Cardiac function was detected by echocardiography using a 2-dimensional guided M-mode ultrasound system for each group. (**c**) The representative heart HE-stained sections were shown for each group (magnification: 20 X). (**d**) Myocardial histopathological scores. (**e**) The survival rate of mice was observed for 28 days following a lethal dose of CVB3 (5LD50) infection. (**f**) Myocardial viral load. Individual experiment was repeated 3 times with 8 mice per group. **P < 0.01.

To determine if C-VP1 immunization has protective effects, 2 weeks after the last immunization, mice were intraperitoneally infected with 3LD50 CVB3 to induce acute myocarditis. Seven days post-viral infection, the severity of myocarditis was evaluated by body weight loss, ventricular systolic function as well as myocardial histological observation. As shown in Fig. 5a, tiny change of weight loss was observed in C-VP1 immunized group. *In vivo* ventricular systolic function was measured by fractional shortening (FS) and ejection fraction (EF) using an echocardiography assay. Compared with L-VP1 immunized group, the left ventricular ejection fraction (LVEF) in C-VP1 immunized mice was ~23% higher and left ventricular fractional shortening (LVFS) was ~19% higher, indicating that *in vivo* ventricular function after infection was improved by prior immunization with C-VP1 (Fig. 5b). Consistently, histological analysis of HE-stained heart sections showed massive myocardial inflammatory infiltration and necrosis in the PBS control group, while moderate or tiny inflammation was seen in L-VP1or C-VP1 immunized mice (Fig. 5c). The lower pathological score was also observed in C-VP1 immunization group compared to L-VP1 immunization group (Fig. 5d). When receiving lethal dose (5LD50) of CVB3 challenge, all mice in PBS-immunized group died within 7 days, while 40% of L-VP1 and 60% of C-VP1immunized mice survived to 28 d with no signs of illness (Fig. 5e). This enhanced protection of C-VP1 seemed correlated with its efficient virus clearance (Fig. 5f). These data indicated that C-VP1 exhibited more efficient immune protection against CVB3-induced myocarditis.

## Discussion

Here, we investigated the effectiveness of immunization of backbone cyclized VP1 protein in providing protection against CVB3 and prevention of viral myocarditis in mice. It was demonstrated that immunization of C-VP1 can dramatically augment the CVB3-specific humoral and cellular immune responses, and a DC maturation was promoted which consequently led to an enhanced protection against CVB3 induced myocarditis.

Although PTS offers a good option for the production of backbone-cyclized polypeptides, this approach needs three main factors to successfully produce circular proteins: first, it would require the proper formation of the three-dimensional structure of intein; second, the termini of target proteins should be in reasonably close proximity and surface exposed in their folded structures^23^,^34^; third, most split inteins require specific amino acid residues at the intein-extein junctions for efficient protein splicing^35^. All factors make it in principle possible to produce circular proteins efficiently *in vivo* in a bacterial expression system. In this study, we demonstrate that the engineered split-intein *Rma DnaB* is efficiently of *in vivo*-cyclizing the VP1 protein of CVB3 in *E. coli*. Optimal expression of the precursor protein RB_C_-VP1-RB_N_ was efficiently *trans*-spliced at 37 °C for 4 h and no precursor protein was detected judging by Western blotting. It was the first report that 100% of the target protein was able to cyclize (described in the Supporting Information). The successfully cyclization of VP1 protein may contribute to its three dimension structure, which shows that the N- and C-termini of VP1 protein are in reasonably close proximity and surface exposed in their native structures, which is amenable for cyclization [21, 22]. Also, the engineered split-intein *Rma DnaB* is proved to be a highly efficient PTS system for protein backbone cyclization *in vivo*, which can be further employed to cyclize small peptides and large proteins. Previously study, several inteins have been used for *trans*-splicing *in vivo* and *in vitro*, such as the *RecA* intein of Mycobacterium tuberculosis^33^,^36^, the *DnaB* intein from *Synechocystis sp. PCC6803 (Ssp DnaB*)^21^,^37^ and *Synechocystis sp. PCC6803 DnaE split* intein (*Ssp DnaE*)^34^,^38^, the maximum reported splicing efficiencies were between 40% to 75% of the protein of interest and the *in vivo* cleavage was often observed.

It has been demonstrated that the cross-linking of the protein termini close to each other by short loop regions would not be expected to cause any changing the overall conformation and activity of the protein^21^. In addition, they are expected to have lower entropy in the unfolded state and should therefore be more stable^20^. Moreover, lacking free termini, they are resistant to exopeptidases. From our results, we could see that the backbone cyclization did not change the overall three-dimensional structures of VP1 protein. Besides, we also demonstrated that the circular VP1 was more stable than the linear VP1 against heat precipitation and exhibited less susceptible to attack by exopeptidase *in vitro*. Furthermore, the protein degradation rate of C-VP1 at the sites of immunization was much more slowly than L-VP1 *in vivo*. In accordance with the stabilization effect of the polypeptide backbone cyclization, mice immunized with C-VP1 induced higher titers of CVB3 specific serum IgG. Moreover, compared with L-VP1, C-VP1 remarkably increased the proliferation and IFN-γ-producing ability of splenocytes T cells. There was also significant difference between mice immunized with C-VP1 and L-VP1 on CVB3-specific CTLs activities of splenocytes in mice. Consistent with the induction of both strong cellular and humoral immunity, mice immunized with C-VP1 significantly reduced myocardial virus dose and led to enhanced resistance to CVB3-induced myocarditis. To obtain a more detailed understanding of C-VP1 mediated immune enhancement, we tested the dendritic cell maturation of immunized mice. DC, the most potent antigen-presenting cells, plays a key role in the induction of the adaptive immune response. Specifically, they are able to uptake and process of exogenous antigens to naive T cells, initiating antigen-specific immune responses. In our results, we observed that C-VP1 immunization significantly improved the maturation of spleen DC through the up-regulation of cell surface expression of MHCII, CD80 and CD86. It could be clearly demonstrated that through the immunization of C-VP1, the immune system is strongly and long-lastingly activated to extent than that caused by L-VP1 that mice are better protected against subsequent lethal infections. As a vaccine, safety is the most concern things. In this study we also tested the tolerance of mice for repeating dosing of C-VP1 (described in the Supporting Information), and the result indicated that the immunization with C-VP1 does not have toxic effect in mice.

In conclusion, several features make C-VP1 as an ideal vaccine candidate for CVB3 infection. First, C-VP1 was produced in *E. coli* under native conditions without costly chemical and multistep manipulation. Second, being a recombinant protein, C-VP1 is an immunodominant structural protein containing several B- and T-cell epitopes. Third, based on concerns over safety, recombinant C-VP1 protein vaccine is only containing purified antigen with defined structure and the degradation products are simply amino acids. Fourth, increased resistance to exopeptidase digestion and improved stability *in vitro* and *in vivo* make backbone cyclization VP1 protein as an attractive recombinant protein vaccine or therapeutic protein. Furthermore, these results suggest the potential of the C-VP1 for use as a vaccine against CVB3 infection and similar approaches could be employed to prepare recombinant protein vaccines to enhance their protection effect.

## Methods

### Construction expression plasmids of circular and linear VP1

The coding sequence of *Rma DnaB* I_N_ and *Rma DnaB* I_C_ was isolated from the engineered *Rma DnaB* mini intein. To construct *Rma DnaB* mini-intein plasmids, inverse PCR was used as previously described^39^,^40^ to replace the putative coding sequences of endonuclease domain with a linker sequence (SAGHHHHHHGGSGS) at the site of deletion. The DNA sequence encoding the C-terminal 43-residue segment (I_C_) followed by two residues (SA) was amplified from *Rma DnaB* mini intein with primers 5′-CATATGGCTGCTCAGAGCGATGTC-3′ and 5′-ACCGGTGGTCTCGCCAGCGCTGTTATGAGCGATATTGTCGTTGG-3′, followed by digestion with *NdeI* and *AgeI*. The N-terminal 104-residue segment (I_N_) of the *Rma DnaB* intein with two extein residues (GG) at its N terminus was amplified by annealing oligonucleotides 5′-ACCGGTGCACCTGCTTCTGGCGGTTGCCTTGCCGGAGACACCCTG-3′ and 5′-CTCGAGTCAGCTAGCCAGTACACGAGGAATACG-3′, followed by digestion with *AgeI* and *XhoI*. The two PCR fragments were cloned between the *NdeI* and *XhoI* sites into the pET-32a vector (Novagen) with a linker sequence, resulting in the plasmid pERB_C_-RB_N_ (Fig. 1b). This linker DNA sequentially contains a *BsaI* and *BfuAI* sites.

The VP1 gene of CVB3 (Nancy strain) was amplified by PCR from pcDNA3.1-VP1^9^ followed by digestion with *BsaI* and *BfuAI* and cloned into the *BsaI/BfuAI* sites in pERB_C_-RB_N_. The resulting plasmid, pERB_C_-VP1-RB_N_ (Fig. 1b), expressed the RB_C_-VP1-RB_N_ fusion protein which the VP1 coding sequence with a histidine at C terminus is in frame with the upstream of *Rma DnaB* C-terminal coding sequence and downstream of *Rma DnaB* N-terminal coding sequence (Fig. 1c). To construct the expression vector of linear VP1, the gene of VP1 was amplified by PCR followed by digestion with *BamHI* and *HindIII*. The PCR product was cloned into the vector pET32a between the *BamHI* and *HindIII* sites, resulting in the plasmid pET–VP1.

### Expression of recombinant VP1 proteins

Competent *E. coli* BL21 (DE3) cells were transformed with pERB_C_-VP1-RB_N_ and pET–VP1, respectively. The cells were grown by continuous shaking at 37 °C, in LB medium containing 100 μg/ml ampicillin until OD≈0.8. Protein expression was then induced by adding IPTG to a final concentration of 1 mM and the incubation was extended for an additional 4 h at 37 °C. The cells were lysed by sonication and the target protein expressed as inclusion bodies. The cyclized and linear VP1 inclusion body proteins were purified and refolded as described previously^41^. The solubilized VP1 from inclusion bodies were refolded by rapid dialysis. In order to get wild type form of linear VP1, the recombinant expressed VP1 protein from pET–VP1 was digested by enterokinase (Sigma) at 4 °C for overnight to remove the fusion tag at VP1 N terminus, then the mix was incubated with Ni-NTA beads (GE Healthcare) and collect the flow-through to obtained purified linear VP1 (L-VP1). Protein concentration was measured by Micro BCA Protein Assay Kit (Thermo) and protein purity was assessed by SDS-PAGE stained with Coomassie blue. Western blot was used for detection VP1 proteins by using anti-VP1 antibody (Dako).

### Re-linearization of circular VP1

The amino acid sequence of VP1 was analyzed by Peptide Cutter (http://web.expasy.org/peptide_cutter/) and it predicted two thrombin-specific cleavage sites in it (Fig. 1c). The purified C-VP1 was incubated with thrombin (Sigma, 1:1000 wt:wt ratio of thrombin to target protein) at room temperature for 16 h to generate linear VP1 (L’-VP1).

### Thermostability and exopeptidase-resistance test

The purified protein solutions were incubated at various temperatures (from 70 to 90 °C). The insoluble fraction was removed by centrifugation at 12,000 × g for 30 min. The supernatants were mixed with SDS-loading buffer and analyzed by SDS-PAGE gel. Carboxypeptidase Y (Sigma) was used to test the exopeptidase-resistance of circular and linear VP1. The purified proteins were digested by carboxypeptidase Y (1:100, carboxypeptidase Y: protein mass-to-mass ratio) at 25 °C, pH 7.5, for 12 h. All samples were analyzed by SDS-PAGE gel.

### Circular Dichroism spectra of VP1 proteins

To investigate the secondary structure of circular and linear VP1, CD spectra of purified L-VP1 (3.6 uM) and C-VP1 (2.2 uM) protein in 10 mM sodium phosphate buffer at pH 8.4 were recorded using J-815 CD Spectrometer (Jasco) in the wavelength range of 190–260 nm at 25 °C. The photomultiplier voltage read never exceeded 600 V in the spectral regions. Each spectrum was scanned three times and the average spectrum was plotted. The cuvette path length was 1 mm for far-UV region measurements with a step size of 0.5 nm and a bandwidth of 1 nm.

### Mice

Six to eight-week-old, male BALB/c mice were purchased from the Experimental Animal Center of Chinese Academy of Science (Shanghai, China). All animal experiments were performed according to the Guide for the Care and Use of Medical Laboratory Animals (Ministry of Health, China, 1998) and with the ethical approval the guide of Soochow University.

Mice were distributed randomly into 3 groups: C-VP1, L-VP1 and PBS. The three groups were immunized intramuscularly with 25 ug of purified recombinant C-VP1, L-VP1 or PBS (as a control) as a 1:1 emulsion with Freund’s adjuvant (FA) 3 times at 2 weeks intervals. Freund’s complete adjuvant (FCA) was used in the first immunization and Freund’s incomplete adjuvant (FIA) was used for subsequent booster injections. Experimental groups consisted of a minimum of five mice, and experiments were repeated at least twice and usually three or four times.

### ELISA measurement of CVB3-specific antibody

Serum samples were collected at 2 weeks after the third immunization and used for evaluation of antibody. Plates were coated with 10 μg/ml VP1_237–249_ peptide (GL Biochem Corp, Shanghai) at 4 °C overnight. After blocking with 5% non-fat milk in PBS, two-fold serially diluted serum were added and incubated at 37 °C for 2 h. After washing the plates three times, HRP-conjugated goat anti-mouse IgG (Southern-Biotech) was added, followed by TMB substrate addition. Absorbance at 450 nm was measured in a microplated reader (Bio-Lab). All serum samples were tested in duplicate.

The avidity of serum IgG was determined by ELISA with a urea-elution step as previously described^42^. Briefly, serum samples (in two fold dilution) were tested in duplicate plates. In one of the plates, 6 M urea (Sigma) was added after incubation with samples and incubated for 10 min. Results are expressed as avidity index calculated as: [(end-point titer in the presence of urea)/(endpoint titer in the absence of urea)] × 100.

### Lymphocyte proliferation assay and ELISPOT assay

Two weeks after the final immunization, mice were euthanized via decapitation. Single-cell suspensions of splenocytes cells were obtained by gentle mechanical disaggregation through sterile 100-um nylon cell strainers (BD Falcon). For the lymphocyte proliferation assay, cells were added to 96-well flat-bottomed tissue culture plates at 5 × 10^5 ^cells/well and stimulated with VP1_237-249_ protein (10 μg/ml). Plates were cultured at 37 °C in a humidified incubator with 5% CO_2_. Three days later, cell proliferation was performed using an ELISA BrdU-Kits (Roche Diagnostics, Mannheim, Germany) according to the manufacturer’s instructions.

IFN-γ producing cell frequency in spleens was assessed using IFN-γ ELISPOT kit (BD Pharmingen). Briefly, cells were plated at 1 × 10^6 ^cells/well and stimulated with VP1_237-249_ protein (10 μg/ml) for 48 h at 37 °C with 5% CO_2_. After sequential incubation with biotinylated detection antibody, streptavidin-HRP and AP-colorimetric substrate, color was developed and spot-forming cells were enumerated with an ImmunoSpot Series 3 Analyzer (Cellular Technology).

### CTL activity assay

Two weeks after the final immunization, splenocytes cells were retrieved from immunized mice and co-cultured with inactivated CVB3 for 3 days and used as effector cells. The plasmid pcDNA3.1-vp1 stably transfected autologous SP2/0 cells were used as target cells. A nonradioactive cytotoxic T lymphocyte assay (CTL) was performed using a lactate dehydrogenase cytotoxicity detection kit (Roche) according to the instructions. Briefly, effector cells and target cells were titrated in U-bottom 96-well tissue culture plates at the E/T ratio as 50:1, 25:1, and 12.5:1, and then 1 × 10^4^ target cells were added. After incubating at 37 °C for 4 h, 50 μL of cell supernatant per well was removed and transferred into the corresponding wells of a 96-well plate. Reaction mixture (50 μL) was added to each well and incubated for 30 min at room temperature. Then absorbance of the samples at 450 nm was measured. The percentage cytotoxicity of CTL was calculated as follows: Cytotoxicity (%) = [(effector and target cell mix–effector cell control) – low control]/(high control–low control)] × 100%.

### Immunofluorescence staining of VP1 protein *in vivo*

Mice were immunized with 25 ug recombinant protein by intramuscularly, then muscles of the immunization site were dissected at day 4 post the immunization and fixed in 10% phosphate-buffered formalin, paraffin embedded. Eight-micrometer frozen sections were prepared and stained with mouse anti-VP1 antibody (Dako) followed by AF680 anti-mouse IgG secondary antibody (Jackson).

### Flow cytometry

For the detection of DC maturation, spleen cells were isolated and centrifuged for 5 min at 2000 g, then stained with a DC myeloid marker, FITC conjugated anti-mouse CD11c (Biolegend), and the following maturation markers (Pecy7 conjugated anti-mouse MHCII, PerCP conjugated anti-mouse CD80, APC conjugated anti-mouse CD86; Biolegend). The percentages of CD11c^+^ MHCII^+^, CD11c^+^ CD80^+^ and CD11c^+^ CD86^+^ cells were determined by flow cytometry using a BD FACS Canto^TM^II instrument. All data were analyzed using FlowJo software version 7.6.

### Challenge with CVB3 virus and evaluation of myocarditis

Two weeks after the final immunization, six mice of each group were infected by intraperitoneally with 3 × 50% lethal dose (3LD_50_) CVB3. After seven days later, assessment of cardiac function was performed using high-resolution ultrasound imaging system (Vevo2100, Visual Sonics, Toronto, Canada) equipped with a 30-MHz microscan transducer. The echocardiographic measurements of left ventricular ejection fraction (LVEF) and left ventricular fractional shortening (LVFS) were performed according to the operator’s manual. Then all mice were killed and the heart was collected from each mouse. Heart tissues were fixed in 10% phosphate-buffered formalin, paraffin embedded, sectioned and stained with H & E. The Histopathological changes were compared quantitatively by calculating the histopathological scores. Two independent researchers individually scored the samples in a blinded manner. For survival rate observation, each group contained eight mice were challenged by 5LD_50_ of CVB3 to observe the percentage of animals surviving during a period of 21 days. Experiments were repeated three times.

### Quantization of viral burden in heart

Seven days after 3LD50 CVB3 challenge, hearts were collected, weighed and homogenized in 10% FBS-RPMI 1640. Samples were serially diluted in 10-fold increments, and incubated on confluent Hela monolayer cells for 7 days to allow plaque formation. Viral titer calculations were determined according to the formula of Reed and Muench and expressed as the logarithm of TCID50 per 0.1 g of tissue.

### Statistical analysis

Statistical analysis was performed with ANOVA followed by Tukey’s post hoc test. Survival rates were analyzed by Kapla-Meier test using GraphPad Prism version 5.01 (GraphPad Software Inc.). Data in Fig 2 are given from a representative experiment of three independent experiments and data in Figs 3, 4 and 5 are given as means ± SD of three independent experiments. P < 0.05 was considered statistically significant.

## Additional Information

**How to cite this article**: Qi, X. and Xiong, S. Intein-mediated backbone cyclization of VP1 protein enhanced protection of CVB3-induced viral myocarditis. *Sci. Rep.* **7**, 41485; doi: 10.1038/srep41485 (2017).

**Publisher's note:** Springer Nature remains neutral with regard to jurisdictional claims in published maps and institutional affiliations.

## Supplementary Material

Supplementary Information
