# Supplementary material for: Intein-mediated backbone cyclization of VP1 protein enhanced protection of CVB3-induced viral myocarditis
Source: Sci Rep. 2017 Feb 2;7:41485. doi: 10.1038/srep41485 (PMC5288654; doi:10.1038/srep41485)
Supplement: Supplementary Information [file srep41485-s1.pdf]

Supporting information

Intein-mediated backbone cyclization of VP1 protein enhanced protection of CVB3-induced viral myocarditis

Xingmei Qi and Sidong Xiong*

Jiangsu Key Laboratory of Infection and Immunity, Institutes of Biology and Medical Sciences, Soochow University, Suzhou, Jiangsu 215123, China

Corresponding author:

Sidong Xiong, PhD

Professor and Director

Institutes of Biology and Medical Sciences, Soochow University,

199 Ren-Ai Road, Suzhou, Jiangsu, 215123, P. R. China.

Phone: 86-512-65881255 Fax: 86-512-65881255

Email: sdxiongfd@126.com

The cyclization efficiency of VP1 protein. During protein cyclization, one important parameter that effects the efficacy of cyclization is the in vivo cleavage including the N- or C- terminal cleavage. In this study, we constructed the fusion protein RB_C-VP1-RB_N with molecular weight of 50 kDa. After cyclization, the molecular weight of cyclic VP1(C-VP1) and RB_N is 32 kDa and 13kDa respectively, which is easily separated by SDS-PAGE, while the RB_C (3 kDa) fragment was too small to detected on SDS-PAGE. If N-cleavage happened, the precursor protein RB_C-VP1-RB_N converts into two products RB_C-VP1 (37 kDa) and RB_N (13 kDa); if C-cleavage happened, precursor protein RB_C-VP1-RB_N converts to VP1-RB_N (46 kDa) and RB_C (3 kDa). According to the bands with apparent molecular masses of SDS-PAGE and Western blotting results (Fig 2a in manuscript), we could easily see that the C-cleavage product VP1-RB_N was not visible, so we can exclude the C-cleavage reaction. In order to test if there N-cleavage reaction happened, we mutated the last amino acid of RB_C Asn to Ala, which usually prevents splicing reaction and leads to N-cleavage reaction. However, form the Western blotting result as Fig S1, we could see that there is only one protein band which is the precursor protein according the molecular weight. This result indicated that the precursor protein also didn’t have N-cleavage reaction. Then, we concluded that the cyclization efficiency of VP1 is 100% and there is no cleavage products during the VP1 cyclization in vivo.

a

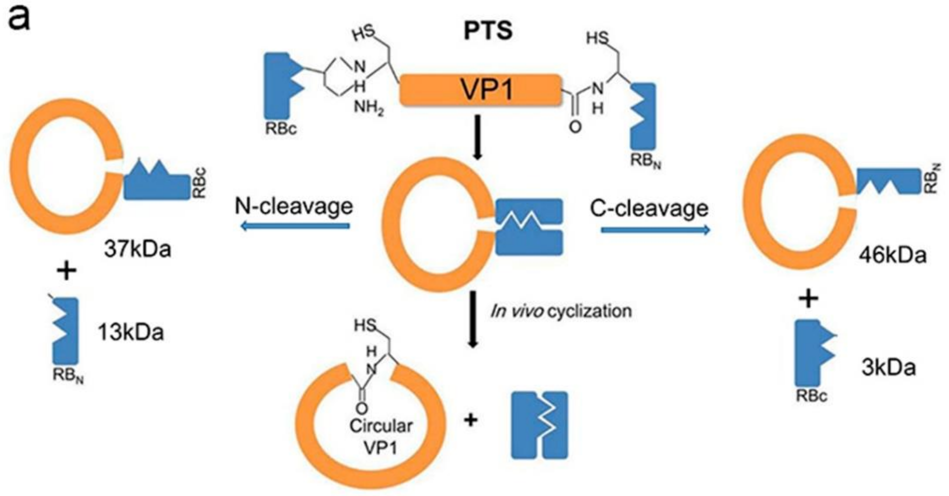

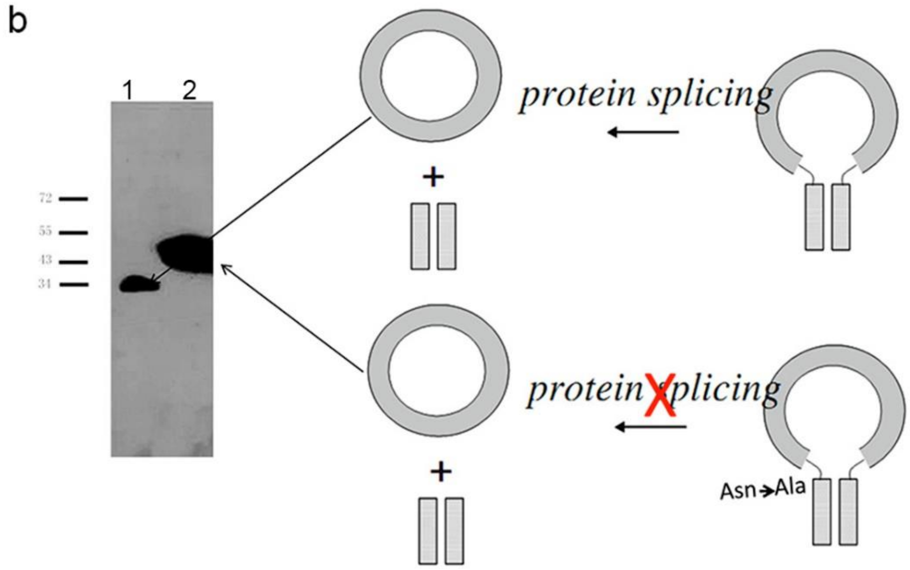

Figure S1. (a) Diagram illustrating of protein cyclization or cleavage. (b) Western blotting analysis of VP1 protein expression, lane 1, VP1 protein cyclization in vivo; lane 2, protein expression after mutation the last amino acid of RB_C Asn to Ala**.**

The tolerance of mice with immunization of C-VP1. In order to test if there toxic effect in mice immunization with C-VP1, three groups of animals were used to ascertain whether repeated dosing of C-VP1 would be well tolerated. Each group has six animals. One group was treated intramuscularly for four days with 100ul of PBS, two other groups were equally treated with 25ug or 100ug C-VP1 suspended in 100ul PBS. The animal body weight and food intake, behavior and aspect were monitored daily. Two days later, mice were euthanized via decapitation. The abdomen was opened and relevant organs were collected including heart, liver, spleen, kidney and intestines. The tissues were fixed in 10% phosphate-buffered formalin, paraffin embedded, sectioned and stained with H & E (Fig S2). The results were shown as follows. In repeated administration, C-VP1 protein were well tolerated by all animals showing no weight loss, normal food intake and no signs of discomfort or abdominal pain were observed

up to 4 days. Also, no histological damages in the heart, liver, spleen, kidney and intestines were observed in C-VP1 administration animals compared to PBS group. These results indicated that immunization with C-VP1 doesn't have toxic effect in mice.

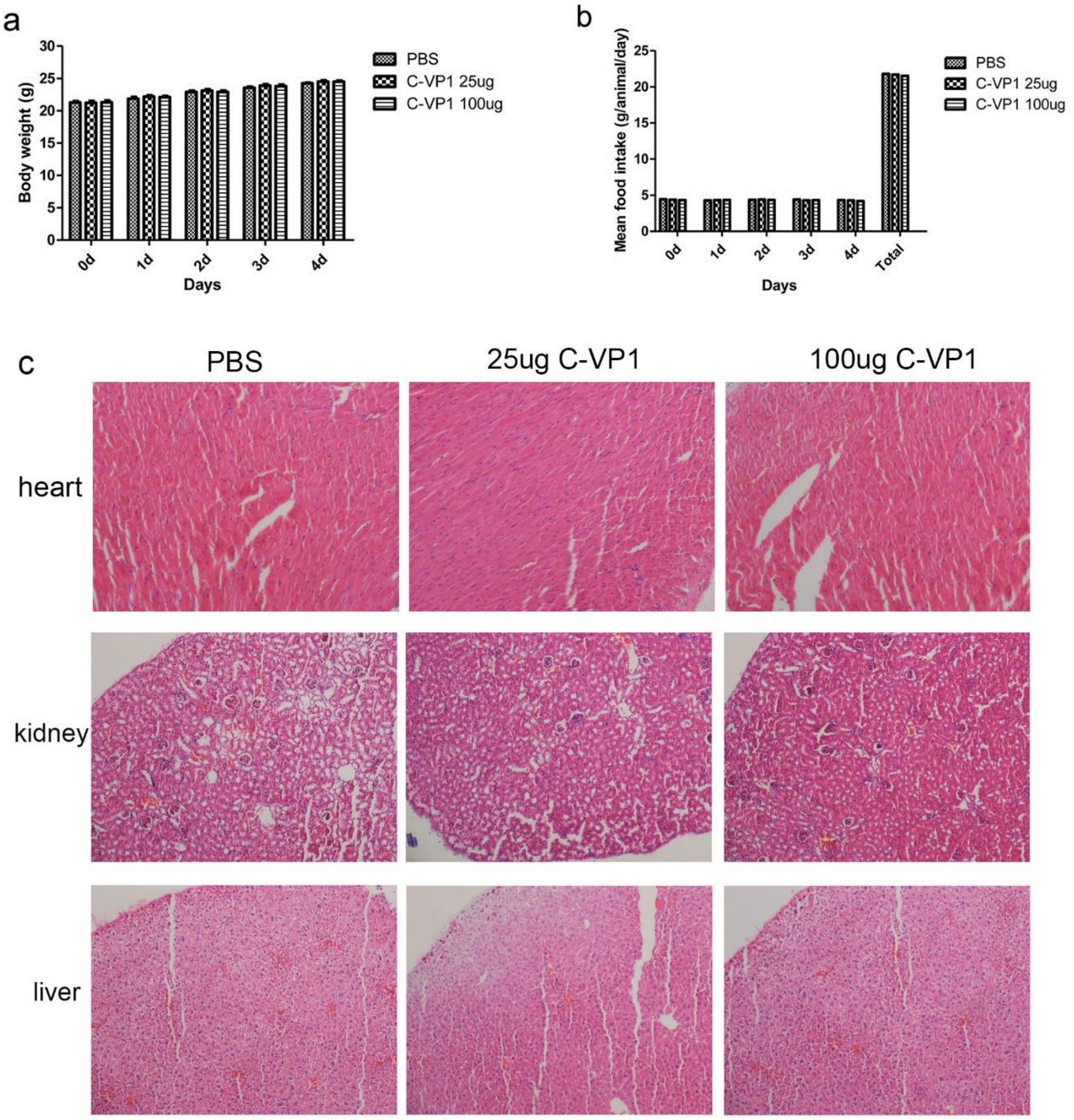

Fig S2. Toxic to mice of immunization with C-VP1. (a) Body weight of animals during intramuscularly administration of C-VP1 compared with control animals. (b) Food intake (per day and accumulated values) of animals during intramuscularly administration of C-VP1 compared with control animals. (c) Histological sections of the organs of C-VP1 administered animals (middle and right) and control animals (left).
